# Preoperative anemia as a marker of postoperative outcomes: a retrospective cohort study

**DOI:** 10.1016/j.bjane.2026.844747

**Published:** 2026-03-28

**Authors:** Liege Caroline Immich, Liliane Ghi Mei Law, Igor Heineck Ouriques, Yan Prates Pimentel, Christian Camatti Menegon, Florentino Fernandes Mendes

**Affiliations:** a Universidade Federal de Ciências da Saúde de Porto Alegre (UFCSPA), Porto Alegre, RS, Brazil; b Universidade Federal de Ciências da Saúde de Porto Alegre (UFCSPA), Graduate Program in Information Technology and Health Management (PGTIGSaúde), Porto Alegre, RS, Brazil; c Universidade Federal de Ciências da Saúde de Porto Alegre (UFCSPA), Post-Graduation Program in Information Technology and Health Management, Department of Surgical Clinics, Porto Alegre, RS, Brazil

**Keywords:** Anemia, Anesthesiology, Elective surgical procedures, Preoperative care, Postoperative complications

## Abstract

**Background:**

Preoperative anemia is common and associated with adverse postoperative outcomes.

**Objective:**

To evaluate the association between preoperative anemia severity and clinical outcomes in patients undergoing elective noncardiac surgery.

**Methods:**

This retrospective cohort included 23,579 adults assessed preoperatively between January 2015 and August 2025, all with documented hemoglobin (Hb) and hospitalized for noncardiac surgery. Patients were stratified as no anemia (≥ 13 g.dL^−1^), mild (11.1‒12.9 g.dL^−1^), moderate (8.1‒11.0 g.dL^−1^), or severe anemia (≤ 8.0 g.dL^−1^). Outcomes were in-hospital mortality, Intensive Care Unit (ICU) admission, and Length of Stay (LOS). Analyses used Poisson regression with robust variance adjusted for confounders.

**Results:**

Among the participants, 15,909 (67.5%) had no anemia, 6,396 (27.1%) mild, 1,174 (5.0%) moderate, and 100 (0.4%) severe anemia. Overall, 62.6% were female, and the mean age was 60.7 years (SD ±15.4). Compared with no anemia, all anemia categories were independently associated with higher in-hospital mortality, increased ICU admission, and longer LOS. Severe anemia was the strongest predictor of in-hospital mortality (adjusted RR = 24.7; 95% CI 13.3‒46.0; p < 0.001). Intermediate or major surgeries (RR = 4.7; 95% CI 3.4‒6.6), age > 54 years (RR = 4.4; 95% CI 2.6‒7.6), and male sex (RR = 2.1; 95% CI 1.6‒2.9) were also independent predictors of in-hospital mortality.

**Conclusions:**

Preoperative anemia, even when mild, was independently associated with higher in-hospital mortality, greater ICU admission, and prolonged hospitalization. These results support systematic screening and targeted management, including Patient Blood Management (PBM) strategies, to improve perioperative outcomes.

## Introduction

Preoperative anemia is highly prevalent worldwide, affecting nearly 30% of patients scheduled for elective surgery, and is recognized as a public health concern by the World Health Organization (WHO).^1^ In the surgical setting, anemia is commonly defined as Hemoglobin (Hb) levels < 13 lt; 13 g.dL^−1^, regardless of sex, due to the expected blood loss and the reduced physiological reserve of surgical patients.^2^, ^3^, ^4^

Previous studies have demonstrated that preoperative anemia increases the risk of transfusion, prolongs the hospital stay, and elevates mortality.^5^ Despite its clinical relevance, most anemic patients do not receive treatment before surgery, representing a missed opportunity for anemia correction and perioperative risk reduction.^6^^,^^7^

In this context, Patient Blood Management (PBM) programs are strongly recommended by international consensus and national guidelines as strategies to optimize anemia management and reduce transfusion exposure.^8^

In Brazil and Latin America, there is still a significant evidence gap regarding the impact of preoperative anemia on surgical outcomes, characterized by data scarcity and high heterogeneity in the implementation of Patient Blood Management (PBM) programs.^9^

Therefore, the objective of this study was to evaluate the association between the presence and severity of preoperative anemia and clinical outcomes in adult patients undergoing elective noncardiac surgery. The primary outcome was in-hospital mortality, and the secondary outcomes were Intensive Care Unit (ICU) admission and hospital Length of Stay (LOS).

## Materials and methods

### *Study design and ethical aspects*

This was a retrospective cohort study conducted in accordance with the STROBE guidelines for observational research. The study was carried out at a tertiary university hospital. The protocol was approved by the Research Ethics Committee of Santa Casa de Porto Alegre (CAAE 83879224.0.0000.5335), with a waiver of informed consent due to its retrospective nature.

### *Population and inclusion criteria*

Adult patients (≥ 18 years) evaluated at the Preoperative Assessment Service between January 2015 and August 2025 were eligible if they had a recorded Hemoglobin (Hb) level and underwent elective noncardiac surgery requiring hospitalization.

**Figure 1:**
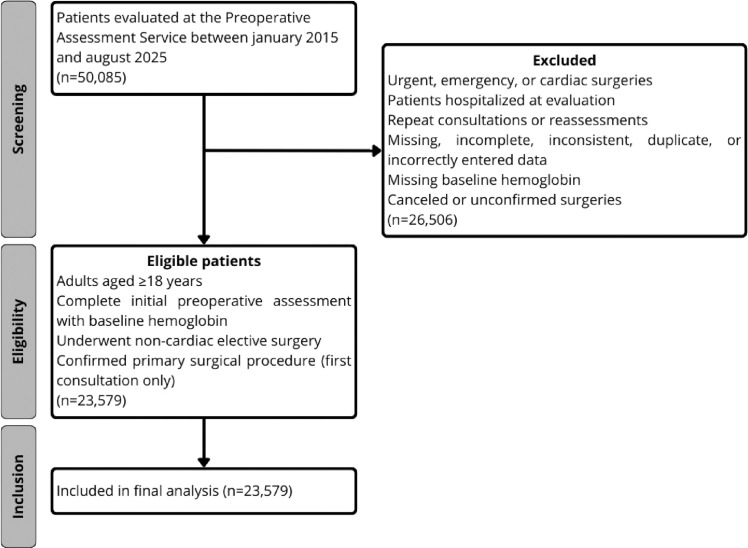
STROBE flowchart of study population identification and enrollment.

To ensure the independence of observations and minimize potential bias, only the first consultation and its corresponding primary surgical procedure were analyzed for each patient; reconsultations and repeat assessments were excluded. The database was queried for patients with a complete triad of initial preoperative assessment, baseline hemoglobin measurement, and confirmed subsequent surgery. Patients with missing laboratory values at the first visit or those whose surgeries were canceled were ineligible and excluded during the screening phase. This selection process is detailed in the STROBE-style flow diagram (Fig. 1). For the overall cohort, the median Hemoglobin-to-Surgery (Hb-S) interval was 26.7-days (IQR 7.9‒90.9). This timeframe supports the clinical relevance and stability of the laboratory findings, as it is consistent with the standard preoperative window for elective procedures and ensures that the reported levels reflect patients’ clinical status prior to surgery.

### *Exclusion criteria*

Patients undergoing urgent or emergency procedures, those already hospitalized at the time of evaluation, cases with incomplete laboratory data, and patients whose surgeries were canceled after evaluation were excluded.

### *Data sources and variables*

Data were retrieved from the institutional electronic health record system (Tasy®) and included demographics, comorbidities, American Society of Anesthesiologists Physical Status (ASA-PS), Body Mass Index (BMI), estimated functional capacity in Metabolic Equivalents (METs), laboratory tests (hemoglobin, creatinine, and glucose), in-hospital mortality, Intensive Care Unit (ICU) admission, and hospital LOS.

Anemia was defined as Hemoglobin (Hb) concentration < 13 lt; 13 g.dL^−1^ for both sexes, following the International Consensus Statement on the peri-operative management of anemia, which recommends this threshold to ensure adequate physiological reserve for surgical stress in all adult patients.^3^ Patients were stratified into four groups based on Hb levels: no anemia (≥ 13.0 g.dL^−1^), mild anemia (11.1‒12.9 g.dL^−1^), moderate anemia (8.1‒11.0 g.dL^−1^), and severe anemia (≤ 8.0 g.dL^−1^). The age threshold of 54 years was selected based on the demographic profile of our surgical population and clinical relevance. This threshold aligns with physiological considerations, as it often marks the post-menopausal period in women ‒ a factor that influences the interpretation of hemoglobin levels and supports the movement toward a unified preoperative hemoglobin target to optimize surgical management. This approach is further supported by the World Health Organization guidance on Patient Blood Management, which emphasizes the need to optimize 'blood health' to improve surgical safety and outcomes.^10^ Creatinine was classified as < 1.5 mg.dL^−1^ or ≥ 1.5 mg.dL^−1^, and glucose was categorized as normal (< 100 mg.dL^−1^), prediabetes (100‒125 mg.dL^−1^), or diabetes (≥ 126 mg.dL^−1^). BMI was classified as underweight (< 18.5), normal weight (18.5‒24.9), overweight (25.0‒29.9), obesity class I (30.0‒34.9), obesity class II (35.0‒39.9), and obesity class III (≥ 40.0). Surgical complexity was categorized as minor, intermediate, or major, based on a previous classification.^11^ Functional capacity was categorized as low (≤ 4 METs) or moderate-to-high (≥ 5 METs), in accordance with clinical standards for preoperative risk assessment.^11^

### *Outcomes*

The primary outcome was in-hospital mortality, while secondary outcomes included Intensive Care Unit (ICU) admission and hospital Length of Stay (LOS).

### *Statistical analysis*

Data were initially entered into Microsoft Excel and subsequently exported to SPSS version 20.0 (IBM Corp., Armonk, NY, USA) for statistical analysis. Continuous variables were described as mean ± standard deviation or median and interquartile range, depending on distribution assessed by the Shapiro-Wilk test. Categorical variables were expressed as absolute and relative frequencies and compared using the Chi-Square test with Yates’ correction when appropriate. Continuous variables without normal distribution were analyzed using the Mann-Whitney test for two groups and the Kruskal-Wallis test with Dunn-Bonferroni post hoc analysis for three or more groups.

The selection of covariates for the multivariable analysis was based on the principle of parsimony and clinical relevance. Variables were included in the final model if they demonstrated a significant association in the univariate analysis or if they were clinically established predictors of postoperative outcomes, such as age, sex, ASA-PS, and surgical complexity. This approach was specifically chosen considering the low-risk profile of the cohort, which is predominantly composed of relatively healthy individuals (ASA-PS I‒II) undergoing minor procedures. In such a population, the potential influence of other clinical confounders or complex comorbidities is expected to be minimal, and a focused model ensures greater stability and avoids overfitting.

Analyses were performed on the entire cohort and repeated on the subgroup of patients undergoing intermediate or major procedures. To adjust for potential confounders, variables selected according to the aforementioned criteria were included in Poisson regression models with robust variance. Relative Risks (RR) with 95% Confidence Intervals (95% CI) were estimated for in-hospital mortality, ICU admission, and hospital LOS. The latter was defined as hospitalization exceeding 4 days, corresponding to the 75^th^ Percentile (P75) of the study population. This threshold was specifically chosen due to the right-skewed distribution of LOS data, ensuring that the P75 accurately represents the clinical boundary of prolonged stay while minimizing the impact of extreme outliers. This approach aligns with established benchmarking methodologies in surgical literature, which utilize the 75^th^ percentile to define reference standards for postoperative outcomes.^12^ A two-tailed p-value < 0.05 was considered statistically significant.

## Results

A total of 23,579 patients with available hemoglobin values and requiring hospitalization were included. The median interval between the preoperative assessment (Hb measurement) and the surgical procedure was 26.7 days (IQR 7.9‒90.9). Of these, 15,909 (67.5%) had no anemia, 6,396 (27.1%) had mild anemia, 1,174 (5.0%) had moderate anemia, and 100 (0.4%) had severe anemia. The cohort comprised 14,768 women (62.6%), with a mean age of 60.7 years (SD ±15.4) (Table 1).

**Table 1:** Baseline patient characteristics by anemia group.

Total sample	Overall cohort (n = 23,579)	No anemia (n = 15,909)	Mild (n = 6,396)	Moderate (n = 1,174)	Severe (n = 100)	p-value
**Age (years)**	60.7 ± 15.4^a^	60.2 ± 15.1^a^	61.2 ± 15.8^a^	64.0 ±1 6.0^b^	58.8 ± 18.2^a^	< 0.001*
**Sex**						< 0.001**
Female	14,768 (62,6%)^a^	8,619 (54.2%)^a^	5,270 (82.4%)^b^	814 (69.3%)^c^	65 (65.0%)^a^^,c^	
Male	8,811 (37.4%)^a^	7,290 (45.8%)^a^	1,126 (17.6%)^b^	360 (30.7%)^c^	35 (35.0%)^a^^,c^	
**ASA-PS**						< 0.001**
I ‒ II	20,353 (87.8%)	14,200 (90.7)^a^	5,370 (85.3%)^b^	740 (64,5%)^c^	43 (44.8%)^d^	
III ‒ V	2,840 (12.2%)^a^	1,453 (9.3%)^a^	926 (14.7%)^b^	408 (35.5%)^c^	53 (55.2%)^d^	
**Procedure complexity**						< 0.001**
Minor	15,515 (65.8%)^a^	10,582 (66.5%)^a^	4,179 (65.3%)^a^	699 (59.5%)^b^	55 (55.0%)^a^^,^^b^	
Intermediate	7,647 (32.4%)^a^	5,065 (31.8%)^a^	2,106 (32.9%)^a^	435 (37.1%)^b^	41 (41.0%)^a^^,^^b^	
Major	417 (1.8%)^a^	262 (1.6%)^a^	111 (1.7%)^a^	40 (3.4%)^b^	4 (4.0%)^a^^,^^b^	
**Comorbidities**						
Hypertension	10,502 (46.4%)^a^	6,699 (43.8%)^a^	3,038 (49.7%)^b^	705 (62.6%)^c^	60 (64.5%)^c^	< 0.001**
DM	3,507 (14.9%)^a^	2,134 (13.4%)^a^	1,072 (16.8%)^b^	273 (23.3%)^c^	28 (28.0%)^c^	< 0.001**
PMI	880 (3.7%)^a^	558 (3.5%)^a^	247 (3.9%)^a^	67 (5.7%)^b^	8 (8.0%)^a^^,^^b^	< 0.001**
Hypothyroidism	1,746 (7.4%)^a^	1,052 (6.6%)^a^	581 (9.1%)^b^	105 (8.9%)^b^	8 (8.0%)^a^^,^^b^	< 0.001**
Hyperthyroidism	100 (0.4%)	64 (0.4%)	32 (0.5%)	4 (0.3%)	‒	< 0.642
CKD	945 (4.5%)	305 (2.1%)^a^	345 (6.1%)^b^	261 (25.3%)^c^	34 (38.6%)^d^	< 0.001**
**Hb-S (days)**	26.7 (7.9‒90.9)	27 (8‒92)	24 (8‒87)	28 (10‒89)	31 (11‒77)	< 0.086***

*Independent *t*-test; **Chi-Square; ***Kruskal-Wallis test.

a,b Different letters indicate statistically significant differences. DM, Diabetes Mellitus; PMI, Previous Myocardial Infarction; CKD, Chronic Kidney Disease; Hb-S, Hemoglobin-to-surgery interval: median and Interquartile Range (IQR).

Patients with anemia had higher in-hospital mortality, increased ICU admission, and longer LOS compared with those without anemia (Table 2).

**Table 2:** Comparisons between patients with and without anemia regarding in-hospital mortality, ICU admission, and LOS in the overall sample and in those undergoing intermediate or major surgery.

Overall sample	No anemia (n = 15,909)	Anemia (n = 7,670)	p-value
In-hospital mortality, n (%)	78 (0.5%)	102 (1.3%)	< 0.001^a^
ICU admission, n (%)	514 (3.2%)	453 (5.9%)	< 0.001^a^
LOS, median (IQR)	1.0 (0.3 – 2.1)	1.0 (0.3 – 2.3)	< 0.001^b^

Intermediate or major surgeries	No anemia (n = 5,327)	Anemia (n = 2,737)	p-value
In-hospital mortality, n (%)	65 (1.2%)	69 (2.5%)	< 0.001^a^
ICU admission, n (%)	452 (8.5%)	368 (13.4%)	< 0.001^a^
LOS, median (IQR)	2.1 (1.0 – 4.1)	2.2 (1.0 – 5.2)	< 0.001^b^

ICU, Intensive Care Unit; LOS, Length of Stay; IQR, Interquartile Range.

a Chi-Square test with Yates’ correction.

b Mann-Whitney *U* test.

Among all patients, 7,647 underwent intermediate and 417 underwent major surgeries, totaling 8,064 individuals. In this subgroup, 5,327 (66.1%) had no anemia, 2,217 (27.5%) had mild anemia, 475 (5.9%) had moderate anemia, and 45 (0.6%) had severe anemia. Of these, 5,015 (62.2%) were women, with a mean age of 63.4-years (SD ±14.6). Again, in-hospital mortality, ICU admission, and hospital LOS were higher in patients with anemia than in those without anemia. These data are presented in Table 3.

**Table 3:** Comparisons of outcomes (in-hospital mortality, ICU admission, and LOS) between patients without anemia and those with anemia (overall and according to severity), in the total sample and among those undergoing intermediate or major surgery.

Total sample	No anemia (n = 15,909)	Mild (n = 6,396)	Moderate (n = 1,174)	Severe (n = 100)	p-value
In-hospital mortality, n (%)	78 (0.5%)^a^	64 (1.0%)^b^	27 (2.3%)^c^	11 (11.0%)^d^	< 0.001*
ICU admission, n (%)	514 (3.2%)^a^	304 (4.8%)^b^	128 (10.9%)^c^	21 (21.0%)^d^	< 0.001*
LOS, median (IQR)	1.0 (0.3 – 2.1)^a^	1.0 (0.3 – 2.2)^b^	1.2 (0.4 – 5.0)^c^	1.3 (0.3 – 8.0)^c^	< 0.001**

Intermediate or major surgeries	No anemia (n = 5,327)	Mild (n = 2,217)	Moderate (n = 475)	Severe (n = 45)	p-value
In-hospital mortality, n (%)	65 (1.2%)^a^	42 (1.9%)^a^	22 (4.6%)^b^	05 (11.1%)^b^	< 0.001*
ICU admission, n (%)	452 (8.5%)^a^	245 (11.1%)^b^	110 (23.2%)^c^	13 (28.9%)^c^	< 0.001*
LOS, median (IQR)	2.1 (1.0 – 4.1)^a^	2.1 (1.0 – 4.4)^b^	3.9 (1.2 – 9.0)^c^	5.1 (0.6 – 14.4)^a^^,^^b^^,c^	< 0.001**

ICU, Intensive Care Unit; LOS, Length of Stay; IQR, Interquartile Range.

*Chi-square; **Kruskal-Wallis test.

a,b Different letters indicate statistically significant differences.

**Figure 2:**
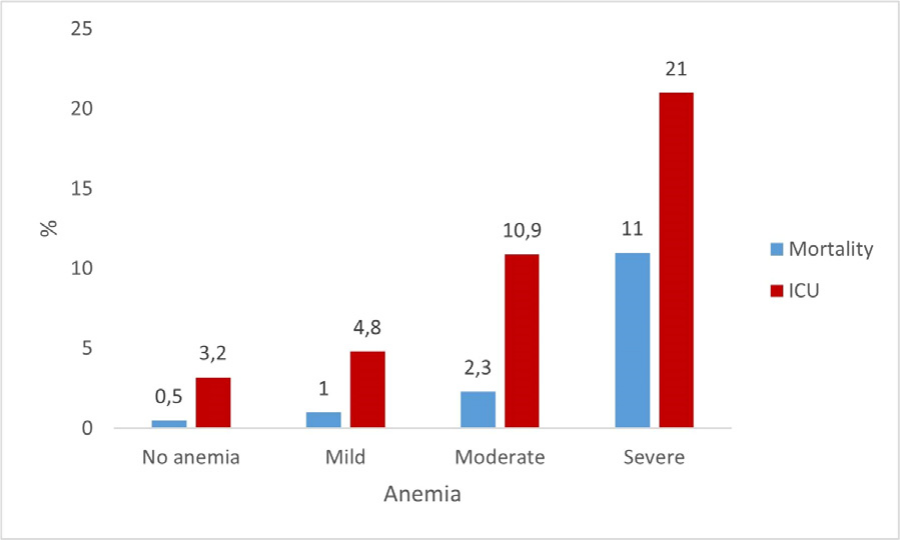
Mortality and ICU admission rates in the overall sample.

In the overall sample, in-hospital mortality and ICU admission were progressively more frequent with increasing anemia severity, with statistically significant differences across all groups. LOS was longer among patients with moderate and severe anemia. In patients undergoing intermediate or major surgeries, in-hospital mortality was higher among those with moderate or severe anemia compared with patients without anemia or with mild anemia. ICU admission was lower in patients without anemia than in those with mild anemia, while patients with moderate or severe anemia had the highest rates. Regarding LOS, patients with moderate anemia had longer stays than those with mild anemia, and those with mild anemia stayed longer than patients without anemia. No significant differences were observed for severe anemia, likely due to small sample size. These results are detailed in Table 3 and illustrated in Figure 2.

Poisson regression models with robust variance were applied to assess factors associated with in-hospital mortality, ICU admission, and prolonged LOS (> 4 days, 75^th^ percentile) in the overall sample and in the subgroup of patients undergoing intermediate or major surgeries. The models were adjusted for potential confounders, including BMI, ASA-PS, hypertension, diabetes, chronic kidney disease, METs, creatinine, sex, age, and surgical complexity.

### *Primary outcome*

Severe anemia had the greatest impact on in-hospital mortality, with a Relative Risk (RR) of 24.7 (95% CI 13.3‒46.0), indicating that severe anemia was associated with a nearly 25-fold increase in the risk of in-hospital death compared to non-anemic patients (adjusted RR = 24.7; 95% CI 13.3‒46.0). Moderate anemia was also significantly associated (RR = 4.6; 95% CI 3.0‒7.1), as was mild anemia (RR = 2.5; 95% CI 1.8‒3.5). Male sex and age > 54 years were also risk factors, as was undergoing intermediate or major surgery compared with minor procedures. When restricted to patients undergoing intermediate or major surgeries, the analysis confirmed anemia severity as a major risk factor. Severe anemia remained the strongest predictor associated with in-hospital mortality (RR = 10.9; 95% CI 4.4‒27.4), followed by moderate (RR = 3.6; 95% CI 2.2‒5.9) and mild anemia (RR = 1.7; 95% CI 1.1‒2.5). Male sex, age > 54 years, and major surgery were also significantly associated with in-hospital mortality. Final model results are shown in Table 4.

**Table 4:** Poisson regression model with robust variance for factors associated with in-hospital mortality in the overall sample and in patients undergoing intermediate or major surgery.

	Overall sample		Intermediate or major surgery subgroup	
Factors	RR (95% CI)	p-value	RR (95% CI)	p-value
**Anemia**				
Severe	24.7 (13.3 – 46.0)	< 0.001	10.9 (4.4 – 27.4)	< 0.001
Moderate	4.6 (3.0 – 7.1)	< 0.001	3.6 (2.2 – 5.9)	< 0.001
Mild	2.5 (1.8 – 3.5)	< 0.001	1.7 (1.1 – 2.5)	< 0.011
No anemia	1		1	
**Sex**				
Male	2.1 (1.6 – 2.9)	< 0.001	1.5 (1.0 – 2.1)	< 0.036
Female	1		1	
**Procedure complexity**				
Major	4.7 (3.4 – 6.6)^a^	< 0.001^a^	4.8 (3.2 – 7.1)	< 0.001
Intermediate			1	
Minor	1		–	–
**Age**				
> 54 years	4.4 (2.6 – 7.6)	< 0.001	3.8 (2.0 – 7.1)	< 0.001
≤ 54 years	1		1	

RR, Relative Risk; 95% CI 95% Confidence Interval.

a Intermediate or major subgroup.

### *Secondary outcome*

In the overall sample, anemia, ASA-PS, sex, surgical complexity, and age were independently associated with ICU admission. Risk increased with anemia severity, as well as with ASA-PS III‒V, male sex, intermediate or major surgeries, and age > 54 years. When restricted to patients undergoing intermediate or major surgery, patients with severe anemia had a three-fold higher risk of ICU admission (RR = 3.0; 95% CI 1.8‒4.9), those with moderate anemia had a two-fold higher risk (RR = 1.8; 95% CI 1.2‒2.8), and those with mild anemia had a 1.4-fold higher risk (RR = 1.4; 95% CI 1.2‒1.6), compared with patients without anemia. Patients classified as ASA-PS III‒V, male sex, undergoing major procedures, and older than 54 years were also at higher risk of ICU admission. The results of the final model are presented in Table 5.

**Table 5:** Poisson regression model with robust variance for factors associated with Intensive care unit admission in the overall sample and in patients undergoing intermediate or major surgery.

	Overall sample		Intermediate or major surgery subgroup	
Factors	RR (95% CI)	p-value	RR (95% CI)	p-value
**Anemia**				
Severe	4.2 (2.9 – 6.3)	< 0.001	3.0 (1.8 – 4.9)	< 0.001
Moderate	2.4 (2.0 – 2.9)	< 0.001	2.3 (1.8 – 2.8)	< 0.001
Mild	1.5 (1.3 – 1.8)	< 0.001	1.4 (1.2 – 1.6)	< 0.001
No anemia	1		1	
**ASA-PS**				
III ‒ V	2.7 (2.4 – 3.1)	< 0.001	2.0 (1.7 – 2.3)	< 0.001
I ‒ II	1		1	
**Sex**				
Male	1.8 (1.6 – 2.1)	< 0.001	1.7 (1.5 – 1.9)	< 0.001
Female	1		1	
**Procedure complexity**				
Major	9.0 (7.6 – 10.8)^a^	˂0.001^a^	3.8 (3.2 – 4.4)	< 0.001
Intermediate			1	
Minor	1		–	–
**Age**				
> 54 years	1.7 (1.5 – 2.1)	< 0.001	1.7 (1.4 – 2.1)	< 0.001
≤ 54 years	1		1	

RR, Relative Risk; 95% CI, 95% Confidence Interval.

a Major or intermediate subgroup.

Prolonged hospital stay (> 4 days) was also evaluated. In the overall sample, severe, moderate, and mild anemia were significantly associated with longer hospitalization. Male sex, intermediate or major surgery, and age > 54 years increased the risk of prolonged stay, while higher functional capacity (moderate-to-high functional capacity category) was protective. In the subgroup of patients undergoing intermediate or major surgery, anemia severity remained significantly associated with prolonged stay. Male sex, major surgery, and age > 54 years increased the risk, while the moderate-to-high functional capacity category reduced it. Results are presented in Table 6.

**Table 6:** Poisson regression model with robust variance for factors associated with LOS (> 4 days, 75^th^ percentile) in the overall sample and in patients undergoing intermediate or major surgery.

	Overall population		Intermediate or major surgery subgroup	
Factors	RR (95% CI)	p-value	RR (95% CI)	p-value
**Anemia**				
Severe	3.0 (2.3 – 3.8)	< 0.001	2.1 (1.6 – 2.8)	< 0.001
Moderate	2.3 (2.1 – 2.5)	< 0.001	1.9 (1.7 – 2.1)	< 0.001
Mild	1.4 (1.3 – 1.6)	< 0.001	1.3 (1.2 – 1.4)	< 0.001
No anemia	1		1	
**METs**				
Moderate-to-high	0.6 (0.6 – 0.7)	< 0.001	0.8 (0.7 – 0.8)	< 0.001
Low	1		1	
**Sex**				
Male	1.8 (1.7 – 1.9)	< 0.001	1.7 (1.5 – 1.8)	< 0.001
Female	1		1	
**Procedure**				
Major	4.5 (4.2 – 4.9)^a^	< 0.001^a^	1.6 (1.4 – 1.8)	< 0.001
Intermediate			1	
Minor	1		–	–
**Age**				
> 54 years	1.7 (1.5 – 1.9)	< 0.001	1.9 (1.7 – 2.1)	< 0.001
≤ 54 years	1		1	

RR, Relative Risk; 95% CI, 95% Confidence Interval.

a Intermediate or major subgroup.

## Discussion

In this study, the presence of preoperative anemia was associated with worse clinical outcomes, including higher in-hospital mortality, increased ICU admission, and prolonged LOS. A severity-dependent association was also observed, characterized by a progressive increase in risk with worsening anemia severity, with severe anemia emerging as the strongest independent predictor of mortality in the adjusted model.

A fundamental and distinct characteristic of our study is the demonstration that preoperative anemia remains a robust marker of adverse outcomes even in a population predominantly composed of patients with low clinical complexity (ASA-PS I‒II) undergoing low-risk surgeries.

While much of the existing literature focuses on high-complexity cohorts – such as cardiac, major oncological, and large-scale orthopedic surgeries ‒ where anemia is a well-recognized complication, evidence shows that the overwhelming majority of these patients still undergo surgery without adequate anemia optimization. Our findings reveal that this systemic failure extends even to routine elective procedures, where anemia remains a clinically significant and frequently neglected risk factor. By investigating a population often underrepresented or diluted in massive datasets, this study provides the clinical specificity necessary to audit current institutional practices. Such contemporary real-world evidence is essential to address the persistent discrepancy between clinical evidence and surgical practice, thereby mitigating the 'therapeutic inertia' that contributes to avoidable postoperative morbidity and mortality. As emphasized in the 2024 World Health Organization Guidance, anemia remains a major global public health challenge, and new data from diverse surgical populations are vital to drive the implementation of Patient Blood Management standards.^10^

Furthermore, it is essential to consider that preoperative anemia may serve as a surrogate marker for broader clinical vulnerability, including advanced comorbidities, chronic inflammation, and frailty syndromes, rather than acting solely as a direct causal driver of mortality. The interplay between low hemoglobin and decreased physiological reserve ‒ often exacerbated by chronic inflammatory states ‒ creates a high-risk surgical phenotype. Although our multivariable models were adjusted for key confounders such as age, ASA-PS, and comorbidities, the retrospective nature of this study limits our ability to fully decouple anemia from these complexes, overlapping syndromes.

Our study distinguishes itself from most of the existing literature by adopting a single hemoglobin threshold of 13 g.dL^−1^ to define anemia for both sexes, rather than the traditional gender-based WHO criteria. This deliberate choice is supported by growing evidence within the Patient Blood Management framework, specifically the International Consensus Statement, which establishes that a Hb concentration ≥13 g.dL^−1^ is necessary to provide sufficient physiological reserve for surgical stress in all adult patients.^3^ The age threshold of 54 years used in our analysis also aligns with these physiological considerations, as it often marks the post-menopausal period in women ‒ a factor that influences the interpretation of hemoglobin levels and supports the movement towards a unified preoperative hemoglobin target to optimize ‘blood health’, a core concept of the 2024 WHO PBM framework.^10^ By applying this stricter criterion, we identified a significant proportion of women who would otherwise be classified as non-anemic by traditional standards, yet who remain at increased risk for adverse postoperative outcomes.

These findings are consistent with international literature. In a UK cohort including more than 39,000 patients, preoperative anemia was identified as an independent predictor of complications and mortality, regardless of other clinical factors.^2^ Similarly, a meta-analysis involving more than 200,000 patients demonstrated that even mild anemia significantly increases the risk of postoperative death.^1^ In orthopedic surgery, a systematic review confirmed that preoperative anemia is associated with higher transfusion risk, prolonged hospitalization, and increased mortality.^5^ More recently, a systematic review with meta-analysis in cardiac surgery reinforced these findings, linking preoperative anemia not only to higher mortality but also to greater morbidity and longer LOS.^13^

Another relevant aspect was the prolongation of hospitalization among patients with anemia, especially in moderate and severe forms. This finding is consistent with prospective studies in colorectal surgery, which demonstrated the feasibility of early preoperative anemia detection and correction, with favorable impacts on recovery.^14^ Regarding ICU admission, since our cohort is predominantly composed of low-to-intermediate risk surgeries where routine ICU use is not standard, admission serves as a proxy for surgical complexity or clinical instability. Importantly, even mild anemia was associated with worse outcomes, underscoring the need for intervention across all severity levels. In our study, higher functional capacity (moderate-to-high functional capacity category) acted as a significant protective factor against prolonged hospital stay, reinforcing the importance of preoperative physical reserve.^11^

The higher rate of ICU admissions among anemic patients identified in this study may be related to reduced physiological reserve, increased vulnerability to infectious complications, and a higher risk of hemodynamic instability. These findings are consistent with international series reporting a higher incidence of infectious and cardiovascular complications in surgical patients with anemia.^1^^,^^2^^,^^5^

In this context, Patient Blood Management (PBM) strategies have gained significant relevance. Current international and national guidelines emphasize the importance of preoperative laboratory screening, etiological investigation, and the specific treatment of anemia before elective surgery.^3^^,^^4^^,^^8^ Intravenous iron supplementation and, in selected cases, the use of erythropoiesis-stimulating agents are effective measures to reduce transfusion requirements and improve outcomes.^6^^,^^7^ Recent evidence from a randomized controlled trial in elderly patients undergoing hip fracture surgery demonstrated that preoperative intravenous iron administration significantly reduced mortality at 6 and 12 months and decreased transfusion needs.^15^ However, it is important to note that hip fracture populations involve urgent orthopedic scenarios, which may differ significantly from the elective noncardiac procedures examined in our study. Differences in surgical urgency, baseline physiological reserve, and the available window for Patient Blood Management (PBM) interventions must be considered when extrapolating these results to elective settings.

In Brazil, a multicenter study reported a high prevalence of preoperative anemia across different regions, regardless of sex and age, characterizing the condition as a public health issue.^9^ Accordingly, the consensus of the Brazilian Association of Hematology, Hemotherapy, and Cellular Therapy recommends postponing elective procedures whenever possible until anemia is diagnosed and corrected, with a minimum of four weeks for treatment.^16^

Our study has limitations that warrant consideration. First, its retrospective, single-center design may limit the generalizability of the findings to centers with different discharge practices or ICU protocols. Second, for the entire study cohort, the overall median interval between Hb measurement and surgery was 26.7 days (IQR 7.9‒90.9). While this interval is clinically acceptable and remained consistent across all anemia severity levels (p < 0.006), changes in Hb levels between the assessment and the procedure were not captured. Additionally, the lack of intraoperative data, such as blood loss and hypotension, precludes a more granular analysis of the surgical insult. Furthermore, the subgroup of patients with severe anemia was relatively small (n = 100); although the association with mortality was profound and clinically significant, the precision of this specific estimate is limited, as reflected by the wider confidence intervals. Finally, while we adjusted for major confounders, the potential for residual confounding from unmeasured frailty or inflammatory markers remains.

## Conclusion

In summary, this study supports the evidence that preoperative anemia is strongly associated with increased surgical mortality, ICU admission, and prolonged LOS. These results reinforce the importance of implementing institutional protocols for screening and treating anemia before elective procedures, in alignment with national and international PBM guidelines, aiming to reduce complications, optimize hospital resources, and improve surgical patient safety. As an observational study, these findings highlight a significant clinical association but do not establish a direct causal link between hemoglobin levels and postoperative outcomes.

## Data availability statement

The datasets generated and/or analyzed during the current study are available from the corresponding author upon reasonable request.

## Declaration of generative AI in the writing process

During the preparation of this work, the authors used Gemini (Google) to refine the English language and improve the structural flow of the discussion. The authors reviewed the final content and accepted full responsibility for the manuscript.

## Declaration of competing interest

The authors declare no conflicts of interest.
